# Supplementary material for: Detection of posttraumatic pneumothorax using electrical impedance tomography—An observer-blinded study in pigs with blunt chest trauma
Source: PLoS One. 2020 Jan 10;15(1):e0227518. doi: 10.1371/journal.pone.0227518 (PMC6953828; doi:10.1371/journal.pone.0227518)
Supplement: Attachment — Submitted filename: Badenes Review PLOS ONE 4.docx [file pone.0227518.s002.docx]

**REVIEWER: Rafael Badenes**

I don´t have any potential biases in reviewing this manuscript.

**Manuscript Number: PONE-D-19-19686**

**Title:** Detection of posttraumatic pneumothorax using Electrical Impedance Tomography - An

observer-blinded study in pigs with blunt chest trauma

**Submitted to:**
PLOS ONE

**1. ABSTRACT.**

- Abstract appropriately summarize the manuscript.

- There aren´t discrepancies between the Abstract and the remainder of the manuscript.

- The Abstract can be understood without reading the manuscript.

**2. BACKGROUND/AIM**

- The Introduction is concise.

- The purpose of the study is well defined.

- The authors provide a rationale for performing the study based on a review of the medical literature with an appropriate length.

- This manuscript is Original Research, with a well-defined hypothesis.

**3. MATERIALS AND METHODS**

*- Please, define and explain more accurate the study design.*

**4. RESULTS**

- The results are clearly explained.

- The results are reasonable and expected.

- There aren´t results introduced that are not preceded by an appropriate discussion in the Methods section.

**5. DISCUSSION**

- The discussion is concise.

- Their research question was answered.

- Authors’ conclusions are justified by the results found in the study.

**6. FIGURES**

- Figure is appropriate and it is appropriately labeled.

- Adequately show the important results.

**7. TABLES (Supl)**

- Appropriately describe the results.

**8. REFERENCES**

- The reference list follows the format for the journal.
