# Supplementary material for: Detection of posttraumatic pneumothorax using electrical impedance tomography—An observer-blinded study in pigs with blunt chest trauma
Source: PLoS One. 2020 Jan 10;15(1):e0227518. doi: 10.1371/journal.pone.0227518 (PMC6953828; doi:10.1371/journal.pone.0227518)
Supplement: Attachment — Submitted filename: Response to Reviewers.docx [file pone.0227518.s003.docx]

**Response to Reviewers**

**Reviewer #1**: Thank you for the opportunity to revise this manuscript. I found this experimental study really interesting, with a robust methodology and an important and clear message, possibly easy to apply in the clinical practice. Also, the authors should be congratulated because of the attempt to find tools able to provide clinical findings at the patients bedside. The manuscript is well written.

I have some minor suggestions:

There are some typo mistakes, please revise the entire manuscript for English and grammar

Introduction: Line 69-70. This information (…may convert to tension pneumothorax..) is redundant

Please add in the introduction some informations about the principles of EIT

The discussion section should be structured according to the order of the results. In this form it is really confusing.

**RESPONSE TO REVIEWER #1**

Thank you for your valuable suggestions. We changed the manuscript accordingly:

- The entire manuscript was revised concerning English and grammar
- The redundant information in line 69-70 was deleted. According, we changed the sentence in line 60 as follows: “Undiagnosed, occult pneumothoraces may convert to tension pneumothorax - particularly after initiating mechanical ventilation - and require emergency thoracostomy (2, 3).”
- As suggested, we inserted some information about the principles of EIT in the introduction
- The discussion section was restructured as suggested

**Reviewer #2:**

1. ABSTRACT.

- Abstract appropriately summarize the manuscript.

- There aren´t discrepancies between the Abstract and the remainder of the manuscript.

- The Abstract can be understood without reading the manuscript.

2. BACKGROUND/AIM

- The Introduction is concise.

- The purpose of the study is well defined.

- The authors provide a rationale for performing the study based on a review of the medical literature with an appropriate length.

- This manuscript is Original Research, with a well-defined hypothesis.

3. MATERIALS AND METHODS

- Please, define and explain more accurate the study design.

4. RESULTS

- The results are clearly explained.

- The results are reasonable and expected.

- There aren´t results introduced that are not preceded by an appropriate discussion in the Methods section.

5. DISCUSSION

- The discussion is concise.

- Their research question was answered.

- Authors’ conclusions are justified by the results found in the study.

6. FIGURES

- Figure is appropriate and it is appropriately labeled.

- Adequately show the important results.

7. TABLES (Supl)

- Appropriately describe the results.

8. REFERENCES

- The reference list follows the format for the journal.

**RESPONSE TO REVIEWER #2**

Thank you also for your helpful suggestions. We changed the manuscript according to your suggestion:

- The study design is defined and explained more accurate in the revised manuscript. The first paragraph of the methods section was changed as follows:

“In compliance with the 3R’s for reduction of animals in research (https://www.nc3rs.org.uk/the-3rs), we used suitable data from an existing institutional database in this experimental, observer-blinded diagnostic study. The original experiment was approved by the governmental animal ethics committee (Landesdirection Leipzig, reference number TVV38/11). Pigs (German Landrace) were received from a conventional pig-breeding farm and were kept at the Large Animal Clinic for Surgery, Faculty of Veterinary Medicine, University of Leipzig in a species-specific environment.

From this database, we randomly obtained 100 EIT files and the corresponding CT data from 17 different pigs. The files were anonymized and observers were blinded to all other information. Two observers (specialists in anesthesiology and intensive care) analyzed the randomly obtained 100 EIT files, while a third observer (specialist in radiology) analyzed the corresponding CT images. “
